# Obesity and the Bidirectional Risk of Cancer and Cardiovascular Diseases in African Americans: Disparity vs. Ancestry

**DOI:** 10.3389/fcvm.2021.761488

**Published:** 2021-10-18

**Authors:** Avirup Guha, Xiaoling Wang, Ryan A. Harris, Anna-Gay Nelson, David Stepp, Zachary Klaassen, Priyanka Raval, Jorge Cortes, Steven S. Coughlin, Vladimir Y. Bogdanov, Justin X. Moore, Nihar Desai, D. Douglas Miller, Xin-Yun Lu, Ha Won Kim, Neal L. Weintraub

**Affiliations:** ^1^Harrington Heart and Vascular Institute, Case Western Reserve University, Cleveland, OH, United States; ^2^Division of Cardiology, Department of Medicine, Medical College of Georgia at Augusta University, Augusta, GA, United States; ^3^Georgia Prevention Institute, Augusta University, Augusta, GA, United States; ^4^Department of Chemistry, Paine College, Augusta, GA, United States; ^5^Vascular Biology Center, Medical College of Georgia at Augusta University, Augusta, GA, United States; ^6^Section of Urology, Department of Surgery, Medical College of Georgia at Augusta University, Georgia Cancer Center, Augusta, GA, United States; ^7^Georgia Cancer Center, Augusta University, Augusta, GA, United States; ^8^Department of Population Health Sciences, Medical College of Georgia at Augusta University, Augusta, GA, United States; ^9^College of Medicine, University of Cincinnati, Cincinnati, OH, United States; ^10^Cancer Prevention, Control, and Population Health Program, Georgia Cancer Center, Medical College of Georgia, Augusta University, Augusta, GA, United States; ^11^Section of Cardiovascular Medicine, Department of Internal Medicine, Yale University School of Medicine, New Haven, CT, United States; ^12^Center for Outcomes Research and Evaluation, New Haven, CT, United States; ^13^Medical College of Georgia at Augusta University, Augusta, GA, United States; ^14^Department of Neuroscience & Regenerative Medicine, Medical College of Georgia at Augusta University, Augusta, GA, United States

**Keywords:** obesity, ancestry, cardiovascular disease, cancer, disparity, cardio-oncology

## Abstract

Cardiovascular disease (CVD) and cancer often occur in the same individuals, in part due to the shared risk factors such as obesity. Obesity promotes adipose inflammation, which is pathogenically linked to both cardiovascular disease and cancer. Compared with Caucasians, the prevalence of obesity is significantly higher in African Americans (AA), who exhibit more pronounced inflammation and, in turn, suffer from a higher burden of CVD and cancer-related mortality. The mechanisms that underlie this association among obesity, inflammation, and the bidirectional risk of CVD and cancer, particularly in AA, remain to be determined. Socio-economic disparities such as lack of access to healthy and affordable food may promote obesity and exacerbate hypertension and other CVD risk factors in AA. In turn, the resulting pro-inflammatory milieu contributes to the higher burden of CVD and cancer in AA. Additionally, biological factors that regulate systemic inflammation may be contributory. Mutations in atypical chemokine receptor 1 (ACKR1), otherwise known as the Duffy antigen receptor for chemokines (DARC), confer protection against malaria. Many AAs carry a mutation in the gene encoding this receptor, resulting in loss of its expression. ACKR1 functions as a decoy chemokine receptor, thus dampening chemokine receptor activation and inflammation. Published and preliminary data in humans and mice genetically deficient in ACKR1 suggest that this common gene mutation may contribute to ethnic susceptibility to obesity-related disease, CVD, and cancer. In this narrative review, we present the evidence regarding obesity-related disparities in the bidirectional risk of CVD and cancer and also discuss the potential association of gene polymorphisms in AAs with emphasis on ACKR1.

Cardiovascular disease (CVD) and cancer often occur in the same individuals, in part because of shared risk factors (1). These risk factors can be classified as modifiable and non-modifiable (2–4). Non-modifiable risk factors include age, gender, genetic variants, and ancestry. Over the years, we have identified that modifiable risk factors- such as smoking, less physical activity, and obesity are independently and separately related to cardiovascular disease and cancer (5, 6). Although significant progress has been made in reducing these modifiable risk factors, rates of obesity continue to rise (7). Obesity contributes to the development of CVD, including atherosclerosis, abdominal aortic aneurysm, heart failure, and certain cancers, such as gastric cancer, through chronic low-grade inflammation (8–10). This inflammatory condition accelerates the onset or progress of carcinogenesis and atherosclerotic plaque formation, thus acting mechanistically as a shared risk factor.

The burden of certain types of CVD such as hypertension, heart failure, and ischemic stroke, and specific cancers such as prostate and breast cancer, as well as cancer-related mortality, is significantly higher in African Americans (AAs) compared to Caucasians (11, 12). At the same time, modifiable shared risk factors exhibit a racial skewing, with a higher proportion of AAs affected by obesity and obesity-associated diseases such as metabolic syndrome (7, 13, 14). Much of this risk can be explained by adverse social determinants of health (SDOH), often abbreviated as socio-economic disparities, that disproportionately afflict AAs, including poor quality diet, lack of safe places to exercise, insufficient access to health care, etc. (15–18).

On the other hand, genetic variability could potentially modify the bidirectional relationship between CVD and cancer. Many genetic variants have been individually linked to both CVD and cancer, but genes that modify the relationship between obesity and the bidirectional risk of CVD and cancer are poorly understood, particularly in AAs. A genetic basis for several diseases that are more common in AAs is well-established. The most noteworthy is sickle cell anemia, a condition resulting from a mutation in the β globin gene (*HBB*) (19). More recently, associations between variants in apolipoprotein L1 (*APOL1*) and chronic kidney disease in AAs have been established, and there is some evidence these variants play a role in increased CVD risk (20). Interestingly, these genetic variants in *HBB* and *APOL1* confer resistance to life-threatening infections common in Sub-Saharan Africa, accounting for their prevalence in AAs (21, 22).

In this narrative review, we focus on the modifiable risk factor of obesity and present the data regarding differences in CVD and cancer risk in AAs compared to Caucasian Americans. We present the current literature on SDOH and its association with CVD and cancer. Additionally, we explore the role of ancestry, specifically a common variant in atypical chemokine receptor 1 [*ACKR1*, also known as the Duffy antigen receptor for chemokines (DARC)], in causation and outcomes in both conditions. Finally, we discuss the translational significance of these risk factors and associations and how this information might be used to identify potential biomarkers for CVD and cancer risk that can be used to help guide personalized therapeutic approaches.

## Obesity as a Shared Risk Factor for CVD and Cancer

Adiposity is traditionally classified based on body mass index (BMI), whereby the normal weight is defined as a BMI 18.5–24.9 kg/m^2^, overweight is defined as a BMI 25–29.9 kg/m^2^, and obesity is defined as a BMI ≥ 30 kg/m^2^ (23). Classes 2 and 3 obesity is defined as a BMI of 35–39.9 kg/m^2^ and ≥40 kg/m^2^, respectively. However, it is important to remember that although BMI is strongly correlated with body fat percentage in population studies, it is limited in its estimation of adiposity on an individual basis and significantly varies based on gender, age, race, ethnicity, and geography (24, 25). It is estimated that up to 50% of the world's population is either overweight or obese (5). In the United States, the prevalence of class 3 obesity is above 7% (7). This prevalence is highly variable across various races and ethnicities, with only a 5.5% prevalence in non-Hispanic white men compared to almost 17% in non-Hispanic Black women (7). It is essential to consider that some of these facts are based on obesity classification using BMI. In contrast, multiple studies have shown that waist circumference and body fat percentage measurements are better measurements of obesity in minority populations (26–28). In a study from the Cancer and Nutrition cohort, mortality increased by 17% in men with the same BMI with a 5 cm increase in waist circumference (29). The study by Kabakambira et al. evaluated African American men and women showed that waist circumference in non-Hispanic Black subjects tracked better with insulin resistance than BMI (27).

### Obesity and CVD

Obesity is estimated to have accounted for over 2.5 million additional CVD-related deaths in 2015 (30). Among individuals with obesity, 41% of deaths, and 34% of disability-adjusted life-years, are caused by excessive weight (10).

The understanding of the association between obesity and CVD has been evolving over the past four decades. It is certainly clear that obesity contributes to the development of atherosclerosis, heart failure, and arrhythmias in different ways (10). The obesity-associated CVD most relevant to this review is atherosclerosis. Obesity is frequently accompanied by other atherosclerosis risk factors, such as hypertension and dyslipidemia, which complicates precise attribution of risk related to obesity, as well as the underlying mechanisms (31). Evidence from research and clinical studies suggests that obesity promotes the development of atherosclerosis by augmenting insulin resistance and inflammation (32–34). Visceral adipose tissue depots are significantly more contributory to this inflammation than subcutaneous fat (35, 36). The liver, in turn, amplifies systemic inflammation by producing large quantities of inflammatory mediators such as various cytokines, complement factors, and C-reactive protein (37). Inflammation plays a key role in multiple aspects of atherosclerosis lesion formation and progression, including uptake of lipid into the blood vessel wall, vascular smooth muscle cell proliferation, vascular remodeling, and lesion destabilization (38). Additionally, endothelial dysfunction caused by an obesity-mediated decrease in endothelial nitric oxide bioavailability is a significant contributing factor (39). While adipose tissues are traditionally considered to contribute to atherosclerosis via indirect (remote) effects on the vascular wall, increasing evidence points toward a pathological role for perivascular adipose tissue, which expands in obesity and becomes more inflamed, thus accelerating vascular disease locally from the “outside-in” (40).

### Obesity and Cancer

Obesity has been identified as a risk factor for several cancers, including a significant number of gastrointestinal cancers (8). The risk varies, with a relative risk of esophageal cancer as high as 4.8 when comparing class 2 obesity to individuals with normal BMI (8). In addition to esophageal cancer, obesity has been associated with increased risk of cancer occurrence for gastric, colorectal, liver, gallbladder, pancreas, breast (postmenopausal), uterine, ovarian, renal-cell, and thyroid cancers (8). By contrast, higher adiposity has been protective in multiple myeloma and meningioma in some studies (8). Nonetheless, 40% of cancer deaths in the US have been attributed to obesity (8, 41).

**Figure 1:**
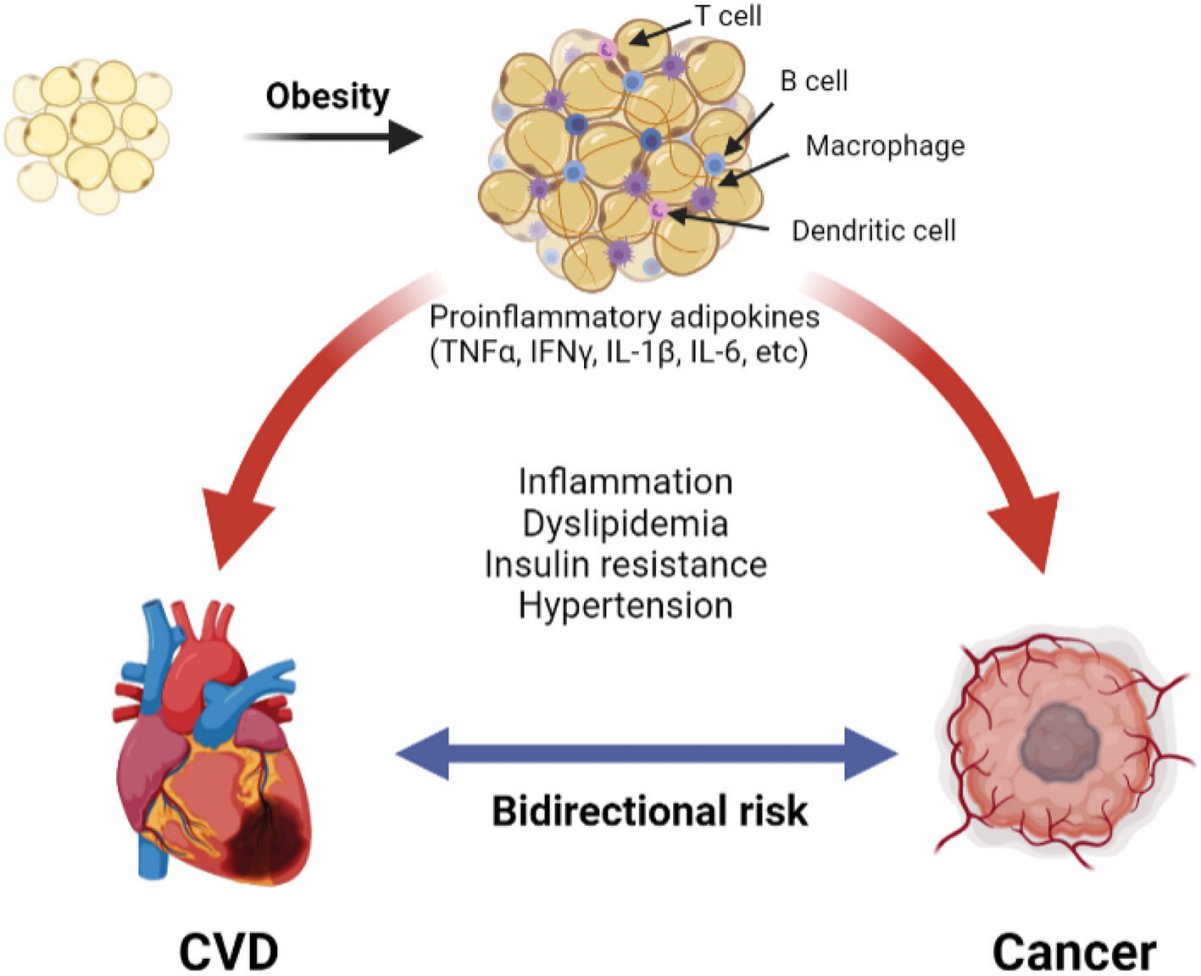
The pathologic adipose tissue microenvironment (ATME) has reduction in vascularity with hypertrophy. This releases damage-associated molecular patterns (DAMP) into the microenvironment, which trigger the infiltration with combination of pro-inflammatory macrophages, dendritic cells, B- and T-cells. Additionally, there is increase in pro-inflammatory cytokines (for example TNFα, IFNγ, etc.). A combination of these creates a chronic pro-inflammatory state that causes cardiovascular disease (31) and cancer (9).

Changes in the adipose tissue microenvironment (ATME) may be mechanistically linked to cancer occurrence in obesity (9, 42, 43). As adipose tissues expand in obesity, adipocyte hypertrophy rather than hyperplasia predominates, and the ATME switches from type 2 [anti-inflammatory; e.g., interleukin (IL)-4, IL-10] to type 1 [pro-inflammatory; e.g., tumor necrosis factor (TNF), interferon (IFN)γ, IL-1β, and IL-6] cytokine generation (42, 44, 45). With further increases in adiposity, the adipocytes become mechanically stressed, and insufficient tissue vascularity leads to ischemia. Damage-associated molecular patterns (DAMPs) released from injured and dying adipocytes further promote infiltration of pro-inflammatory cells, such as dendritic cells and M1-macrophages, leading to the development of crown-like structure (CLS), a pathognomonic feature of adipocyte necrosis (9, 45). Numerous lymphoid cells, such as B-cells and CD8 T-cells, are also observed in the inflamed adipose tissue. This sustained inflammation is associated with a tumor environment that promotes cancer primarily through local rather than systemic stimulation of tumorigenesis, as cancers that are not directly surrounded by adipose tissues have a weaker association with obesity. The above crosstalk between adipocytes and cancer cells promotes tumorigenesis in obesity through complex mechanisms that have been reviewed elsewhere (9) and are summarized in Figure 1.

## Role of Obesity in the Bidirectional Association Between Cardiovascular Disease and Cancer

Obesity-associated inflammation and insulin resistance promote both CVD and cancer. Additionally, CVD and cancer themselves can be developed from proinflammatory states (46, 47). Thus, we can speculate that either CVD or cancer is additive to obesity in promoting the other condition (e.g., those with CVD and obesity are likely at a higher risk of cancer and vice versa). Both epidemiological and mechanistic data demonstrate a potential increase in cancer development in those with CVD.

A Danish national registry study showed that patients with myocardial infarction were 14% more likely to develop cancer (48). Lau et al. using the Framingham 3rd offspring cohort, reported that patients with the highest CVD risk (>20% in 10-years, calculated using the pooled cohort equation) had a 3–4 times higher risk of cancer development than those at the lowest risk (<5% in 10-years) over a 15-year follow-up (49). To confirm these findings experimentally, Meijer et al. used a murine model of colon cancer and demonstrated accelerated cancer growth upon induction of myocardial infarction, and shotgun proteomics identified a number of pro-inflammatory cytokines that were associated with this phenomenon (50). Additionally, the authors confirmed that patients in a population cohort with congestive heart failure had elevated pro-inflammatory cytokine levels. Furthermore, the role of inflammation in cardiovascular disease in promoting carcinogenesis has also demonstrated this phenomenon in murine breast and lung cancer models (51, 52). There is also evidence to suggest that there is a role of the renin-angiotensin-aldosterone system (RAAS) in increasing the risk of cancer in patients with congestive heart failure (53). However, how obesity modifies this mechanistic pathway is to be determined.

### Racial Disparity in Bidirectional Association

A priori, we propose that increased adiposity can alter the rate of cardiovascular aging following the initial cancer diagnosis, which in turn can contribute to greater CVD risk over time. AAs exhibit greater adiposity compared to Caucasians and cancer mortality is also higher in the presence of obesity. Although the role of obesity in modifying the effects observed in these epidemiological or mechanistic studies has yet to be investigated, there is some evidence to support the role of obesity in carcinogenesis and cancer prognosis in AAs. Obesity has been shown to be independently associated with pancreatic cancer in AAs (54). Although obesity seems protective against multiple myeloma (8), obesity contributes to the increased incidence and mortality of multiple myeloma in AAs compared with other population groups (55). Although the role of adiposity in men with prostate cancer is somewhat controversial (56, 57); obesity, in general, can increase the risk of prostate cancer (58, 59). In fact, AA men exhibit more aggressive forms of prostate cancer (60), independent of BMI. In addition, AA women are almost twice as likely to be obese compared to Caucasian women (11) and greater adiposity has been shown to contribute to the increased prevalence of breast cancer (61). In addition, women with greater adiposity exhibit an increased risk of estrogen-receptor-positive (ER+) breast cancer and a decreased risk of triple-negative (TN) tumors (62) compared to average-weight women.

## Obesity in AAs—Role of Disparities and Social Determinants of Health (SDOH)

The relative contribution of obesity to CVD and cancer may depend on the population that is being investigated. Notably, in the United States, AAs are 1.5 times more likely to be obese compared with Caucasians (7). While several factors likely contribute, this trend primarily reflects socio-economic disparities (15, 18). Systemic racism has led to years of redlining in many parts of the country (63). Redlining is defined as the systematic denial of various services or goods by governmental agencies or the private sector, either directly or through the selective raising of prices which disadvantages the poor and minority communities (64, 65). This is a reason why many AAs live in food deserts with low availability of affordable, healthy foods (17, 66). In addition, certain areas have poor access to quality education and primary care physicians, leading to a lack of knowledge about weight management and healthy eating (67–69). Further, lack of access to quality education leads to lower-income and poverty, thereby reducing economic advancement opportunities (16, 70). The cycle of disparities related to these SDOH is self-perpetuating, leading to an exponential increase in obesity (16). It is predicted that one out of 4 AA adults will be severely obese by 2030 (7).

The direct effect of these disparities is reflected in poor cancer and CVD outcomes, particularly in the Southeastern United States, where the proportion of AAs in the rural regions is the highest (12, 71, 72). Although there has been a reduction in racial disparity in cancer mortality, the risk of mortality after cancer diagnosis remains 14% higher in AAs compared to Caucasians (12). Specifically, AA men are 111% more likely to die of prostate cancer when compared to Caucasian men, and AA women are 39% more likely to die of breast cancer compared to Caucasian women (12). AAs also have substantially higher rates of fatal atherosclerotic vascular disease compared with Caucasians [men: hazards ratio (HR), 2.18; women: HR, 1.63] (73).

**Figure 2:**
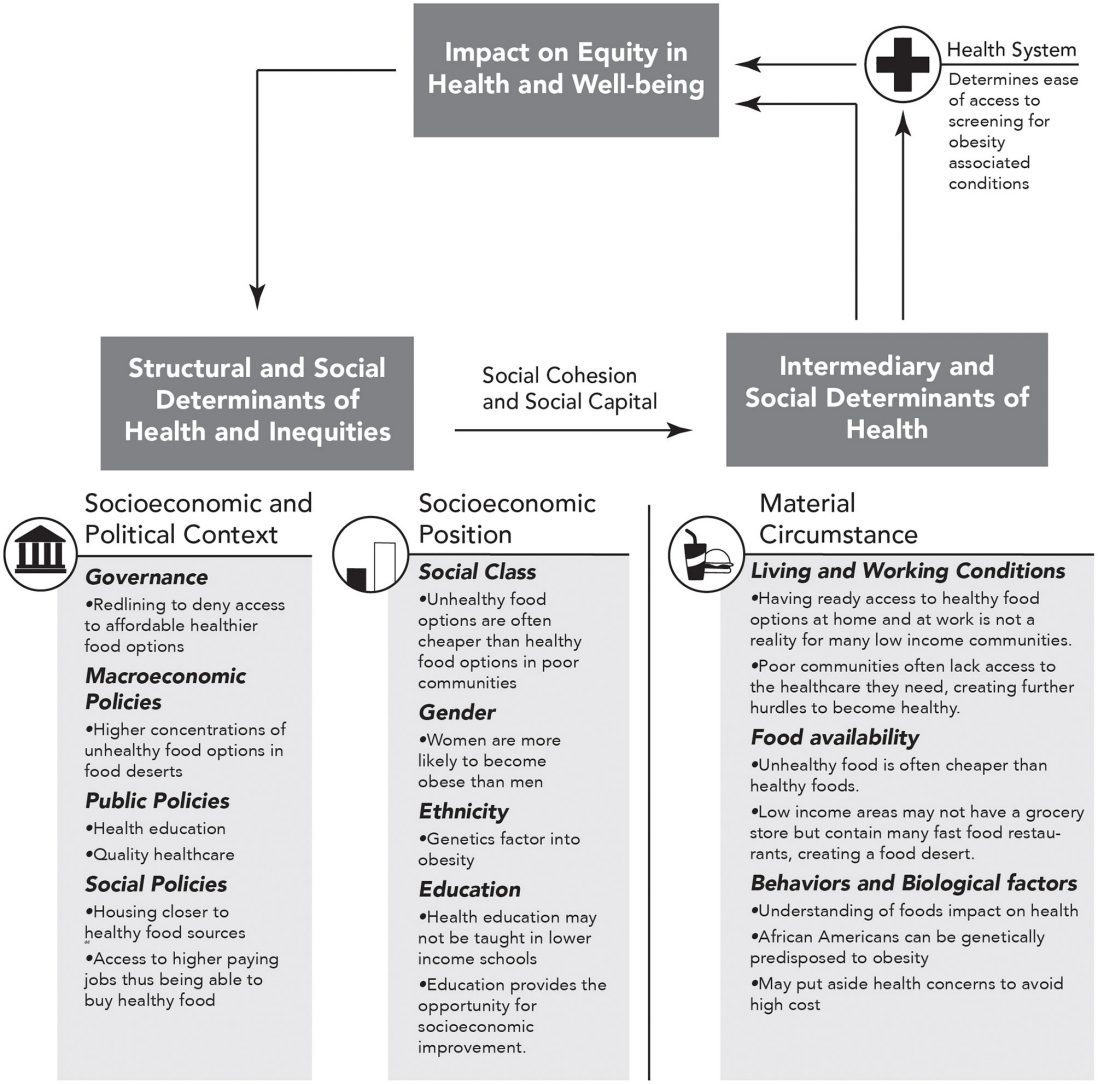
Obesity specific modification of figure A on page 6 of “A Conceptual Framework for Action on the Social Determinants of Health” document (74). Here we show how social determinants of health (SDOH) impact equity in health and well-being and how repetition within this cycle can increase in obesity and thus obesity associated CVD and cancer. The arrows represent directional association.

In order to fully determine the role of adverse SDOH in mediating obesity-related disparities in the bidirectional relationship of cancer and CVD, it is important to objectively assess SDOH in a systematic epidemiological study. The World Health Organization (WHO) established the commission on SDOH (CSDH), which developed a framework with two major components (74). The first element of the framework is the socio-economic and political context. Table 1 summarizes the six aspects of the first element, which includes governance, macroeconomic policies, social policies, public policies, cultural, and societal values as well as epidemiological conditions. The second element of the framework is socio-economic position, which is operationalized by income, education, occupation, social classes, gender, and race/ethnicity. The CSDH framework posits that structural determinants generate or reinforce stratification in society, thus influencing the individual socio-economic position. The first and second elements lead to change in health through intermediary social factors, i.e., the SDOH. The SDOH are classified further into material-, psychosocial-, behavioral/biological-, and health system-related factors. While designing an epidemiological study, it is essential to realize that the SDOH are secondary features that need to be explained due to either the first or the second element. The standard measurements of the SDOH are also presented in Table 1, and the role of SDOH in relationship to obesity is illustrated in Figure 2. It is important to note that several of these indices are worse in AAs when compared to Caucasians.

Table 1The World Health Organization framework for Social Determinants of Health.

**Table:** 

**Structural determinants**			
**Socio-economic and political context**	**Example (ref)**	**Variable selection in population level epidemiological studies**	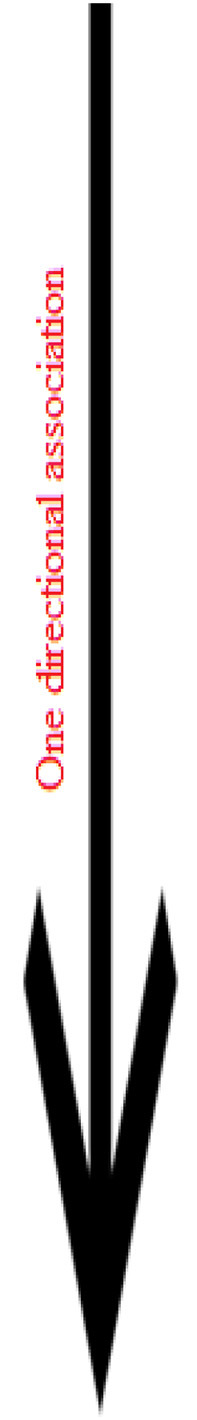
Governance	Welfare State concept (75, 76)	Capitalism vs. Socialist State, Democratic vs. Republican government	
Macroeconomic policies	Unemployment insurance, early retirement (77)	Average age of retirement, % unemployed	
Social policies	Public provision of basic education, health services, and housing (76)	Distance traveled to see primary care doctor	
Public policies	Governmental rules regarding the various contexts above (78, 79)	% GDP spent on healthcare, % GDP spent on equitable housing	
Cultural and societal values	Cultural context and cardiovascular disease (80)	% Of a specific religion	
Epidemiological conditions	Current state of various diseases and risk factors in the area. Eg. HIV (81)	% Smokers, % of area suffering with HIV	
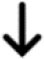 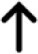 Bidirectional association			
**Socio-economic position**			
Income	Poverty and its effect on health (82)	Median income of household, % living below poverty line, GINI index (83)	
Education	Cancer mortality in the United States by education level (84)	% educated till 10th grade, % college graduates	
Occupation	How occupation contributes to risk factors for cardiovascular disease (85)	Classified into professional, Intermediate, Skilled non-manual, Skilled manual, Partly skilled, Unskilled	
Social classes	Associations of social class with patterns of general and mental health (86)	% Without any vehicle, % veterans, % disabled	
Gender	Gender disparities in the survival of metastatic colon cancer (87)	%Women, %trangender/homosexual	
Race/ethnicity	Racial disparities in use of breast cancer therapy (88)	% African-American, % Hispanic

**Table:** 

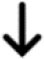 One directional association		
**Intermediary determinant or social determinants of health**		
Material	Effect of housing quality on hypertension (89)	% In neighborhood with refrigerator, telephone, internet, number of people in a household
Psychosocial	Violence and Cardiovascular Health (90)	% With significant debt, marital status, h/o of serious accident
Behavioral and biological	Smoking and cancer (91)	Smoking, diet, alcohol use, exercise
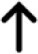 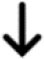 Bidirectional association		
Health system related	Access to quality primary care and coronary artery disease (92)	Number of specialists in the zip code, % compliance with mammography

*This corresponds to figure A on page 6 of “A Conceptual Framework for Action on the Social Determinants of Health” document (74). We present an example from literature with reference along with variable selection in epidemiological studies. ↓ and ↑ Represent directional association. Both elements of structural determinants affect the Social Determinants of Health. Specifically, health system-related social determinants of health are connected to the material, psychosocial, behavioral, and biological contexts*.

## Ancestry; Polymorphisms in AAs

While SDOH undoubtedly accounts for much of the obesity-related disparities in the bidirectional risk of CVD and cancer in AAs, genetic factors can likely modify this relationship. Several genetic factors have been explored for their potential role (11, 93). Here, we will focus on two genes that may be of particular relevance: *APOL1* and *ACKR1* (or DARC).

The APOL1 gene regulates the process of autophagy and cell death, and APOL1 gene polymorphisms are relatively more common in those with West African ancestry (94). Two common polymorphisms, G1 and G2, have been associated with chronic kidney disease (95, 96). These APOL1 gene polymorphisms are located in the serum response associated (SRA) binding region and reduce the ability of SRA to bind APOL1 (94). The polymorphisms are associated with reduced risk for infection from the vector-borne ailment of African sleeping sickness, which is caused by Trypanosoma protozoans endemic to that part of the world, thus explaining their prevalence in the AA population (97). Indeed, as many as 21% of those from West Africa carry the G1 polymorphism, while 13% carry both G1 and G2, which are associated with increased risk of hypertension and renal disease (95, 97). With regard to CVD risk, population studies suggest that individuals with two APOL1 polymorphisms are at increased risk of cardiovascular mortality, surviving on average 3 years less than AAs harboring one or no APOL1 risk alleles (98). Further, the risk of myocardial infarction was estimated to be 80% higher in individuals carrying two of the risk alleles (98). Nonetheless, the mechanisms linking these alleles to CVD risk remain to be determined. Given the biological function of APOL1 in autophagy and cell death, its potential role in regulating cancer growth and outcomes seems more evident. In this regard, surprisingly little has been reported aside from a role in renal cell carcinoma, and a different polymorphism in the gene likely plays a role in this disparity (99). Further investigations into the role of APOL1 in both cancer and CVD are certainly warranted.

Mutations in ACKR-1, otherwise known as DARC, confer protection against malaria and are highly prevalent amongst AAs and other persons of color, including Hispanic Caucasians (100). Notably, ~70% of AAs harbor a T > C mutation at nucleotide−33 (GATA-1 binding site) in the promoter region that silences expression of ACKR-1 on red blood cells (RBC) (101). ACKR-1 is a promiscuous non-signaling chemokine receptor prominently expressed on RBC and to a lesser extent on other cell types such as endothelial cells and adipocytes. On RBC, ACKR-1 is thought to act primarily as a chemokine modulator by sequestering chemokines (i.e., “buffer-sink” function) or by regulating their local concentration at sites of inflammation (102). Among various chemokines, ACKR-1 has a strong binding affinity for CCL2/ monocyte chemoattractant protein (MCP-1), and ACKR-1 negative individuals are more sensitive to MCP-1-induced monocyte mobilization (102–104). Given the vital role that monocytes and inflammatory chemokines play in both CVD and cancer, common ACKR-1 polymorphisms would seem to be obvious candidates to modify the bidirectional risk between cardiovascular disease and cancer in AAs (105–107).

The association between ACKR-1 polymorphisms and inflammation is complex. Global ACKR-1 knockout mice exhibit enhanced susceptibility to diet induced-obesity/adipose tissue inflammation and increased severity of prostate cancer compared with wild-type mice, which is in line with clinical data in African Americans vs. Caucasians (106, 108, 109). Studies suggesting a role for pro-angiogenic ACKR-1 binding chemokines in the pathogenesis of prostate and breast cancer provide a mechanistic basis for how the loss of expression of ACKR-1 on RBC might promote tumor growth (110–112). On the contrary, the same ACKR-1 polymorphism common in AAs is also associated with a reduced number of neutrophils and neutrophil/lymphocyte ratio, a condition known as benign ethnic neutropenia (113). This could potentially function to offset the pro-inflammatory effects of the ACKR-1 gene mutation. Moreover, under some conditions, the absence of ACKR-1 may promote chemokine receptor desensitization, suggesting that loss of ACKR-1 expression may provoke either pro-inflammatory or anti-inflammatory responses on the type and chronicity of inflammation, local environmental factors, etc. There is evidence to suggest that ACKR-1 overexpression reduces the likelihood of metastatic disease in breast cancer (114). Similarly, global DARC knockout mice in the apolipoprotein E knockout background have been shown to exhibit reduced aortic atherosclerosis as compared to their wild-type counterparts (115). While the clinical relevance of this finding is unclear, it is important to point out that coronary artery calcifications, a pathognomonic feature of atherosclerosis, are consistently lower in AAs as compared with Caucasians (116).

## Epidemiological Framework

**Figure 3:**
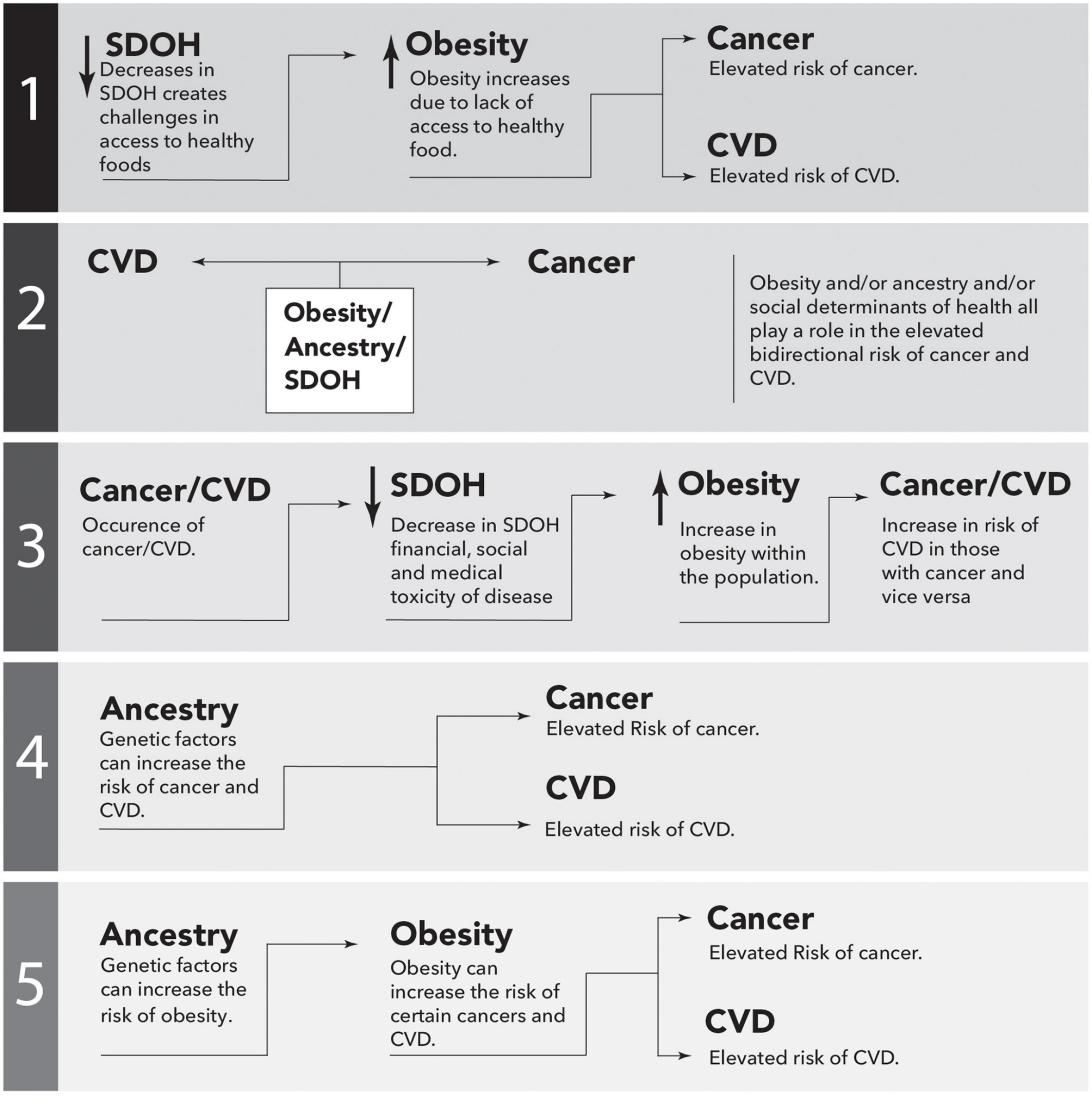
Simplistic causal directed acyclic graphs to illustrate possible epidemiological associations between cancer and cardiovascular disease (CVD). Performing these studies would potentially address the missing links between ancestry, Social Determinants of Health (SDOH), obesity and the bidirectional risk of CVD and cancer. The arrows represent directional association.

Although there is mounting evidence to suggest that obesity is associated with the bidirectional risk of cancer and CVD, the precise level of risk, and the underlying mechanisms, are not fully understood. Moreover, it is apparent that the risk disproportionately affects certain ethnic groups, such as AAs. The SDOH plays a significant role in contributing to these health disparities, and addressing them is necessary to reduce the risk. Additionally, ancestry may contribute to the increased bidirectional risk, resulting in a maladaptive phenotype in AAs potentially due to the interaction of these two parameters. The role of obesity, SDOH, and ancestry should be investigated both independently and together in an effort to understand the relative importance of each of these elements in disease occurrence, disease outcomes, and therapeutic benefit. Examples of studies that would potentially address the disparities are sketched in Figure 3.

## Author Contributions

AG, NW, XW, and RH conceived the study concept. References were collected first by AG and verified by NW. AG drafted the first version of the manuscript. The study was conducted under the supervision of NW. All authors provided necessary revisions of the manuscript.

## Funding

This work was supported in part by American Heart Association-Strategically Focused Research Network Grant 863622 in Disparities in Cardio-Oncology (AG, XW, RH, DS, VYB, and NW). NW was supported by NIH Grants HL124097, HL126949, HL134354, AR070029, and AG064895. RH was supported by Grants DK117365, DK125013, HL137087, CFFT Harris19A. JM was supported by Grant K01MD015304 from the National Institute on Minority Health and Health Disparities of the National Institutes of Health.

## Conflict of Interest

The authors declare that the research was conducted in the absence of any commercial or financial relationships that could be construed as a potential conflict of interest.

## Publisher's Note

All claims expressed in this article are solely those of the authors and do not necessarily represent those of their affiliated organizations, or those of the publisher, the editors and the reviewers. Any product that may be evaluated in this article, or claim that may be made by its manufacturer, is not guaranteed or endorsed by the publisher.
